# PReS-FINAL-2256: Panniculitis revealing quantitative alpha 1 antitrypsine deficiency

**DOI:** 10.1186/1546-0096-11-S2-P246

**Published:** 2013-12-05

**Authors:** A Gagro, N Pustišek, J Čepin Bogović, A Rožman, E Ozimec, AM Kapović

**Affiliations:** 1 Children's Hospital Zagreb, Zagreb, Croatia; 2 Clinic for Respiratory Diseases, Zagreb, Croatia; 3 General Hospital Zabok, Zabok, Croatia

## Introduction

Panniculitis can arise from many underlying causes. Potential causes include connective tissue disease such as systemic lupus erythematosus and scleroderma, lymphoproliferative disorders, pancreatic disease, gout, chronic kidney disease, alpha 1-antitrypsin (alpha1 AT) deficiency and adverse reactions to medications. Alpha 1 AT deficiency is one of the most common hereditary disorders of Caucasians and Europeans, and commonly associated with pulmonary and hepatic injury. Panniculitis is an uncommon first manifestation of this deficiency, with fewer than 100 cases reported in the English-language scientific literature.

## Objectives

We report the case of a 14-year-old boy who presented with recurrent panniculitis on his chest and proximal extremities for 2 years subsequent to trauma after his parkour training sessions. On one occasion the lesion on upper right arm broke down and ulcerates, causing an oily discharge while others regressed spontaneously. Despite treatment with antibiotics the lesions did not improve.

## Methods

The patient was evaluated for possible causes of panniculitis following a biopsy that showed a septal panniculitis with lymphocytes and histiocytes infiltrate within fibrous septi, and around venules.

## Results

Rheumatoid factor, antinuclear antibody (ANA), anti-dsDNA and antineutrophil cytoplasmic antibody (ANCA) were all normal. Alpha-1 antitrypsin level was found to be less than 0.45 g/L on two occasions (ref. 0.9-2.0 g/L). Enzyme genotyping demonstrated a MZ pattern. Pulmonary function testing was normal as was the liver function tests. Evaluation of his family members showed that two members had the same phenotype (father and older sister) but neither of them had a history of panniculitis or other manifestations that could be associated with alpha1 AT deficiency.

## Conclusion

The reported experience suggests that panniculitis occurs equally among men and women with alpha 1 AT deficiency and that the mean age of onset is approximately 40 years old. We report this case so that the condition may be suspected in pediatric patients with panniculitis.

## Disclosure of interest

None declared.

